# Epidemic Dynamics Post-Cyclone and Tidal Surge Events in the Bay of Bengal Region

**DOI:** 10.5334/aogh.4751

**Published:** 2025-07-22

**Authors:** Sajda Khatoon, Paramita Bhattacharya, Nirmalya Mukherjee, Pranay Lal, Martin W. Bloem

**Affiliations:** 1 Centre for Public Health Research-MANT, India; 2 Martin W. Bloem, Johns Hopkins University, USA

**Keywords:** MPCSs, cyclones, climate change, adaptation, Sundarbans, Bay of Bengal

## Abstract

*Background:* The Sundarbans, prone to cyclones and tidal surges, witnessed 13 cyclones (1961–2020), causing widespread water and vector-borne diseases, injuries, deaths, crop and livestock loss, and long-term health issues.

*Objectives:* This study investigates the impact of multi-purpose cyclone shelters on the health outcomes of the Sundarbans population, focusing on epidemic-prone diseases caused by these natural disasters.

*Methods:* The study used secondary data from the Health Management Information System (HMIS) portal, Census of India, International Best Track Archive for Climate Stewardship (IBTracs), Department of Disaster Management, and Environmental Systems Research Institute, Inc (ESRI) India, to understand the association of environmental, social, demographic, geographic, and economic factors on water and vector-borne diseases and cyclonic events for 19 census development (CD) blocks. Maps were prepared using ArcGIS Pro v.2.8. A literature review was undertaken to assess the effectiveness of cyclone shelters and potential shortcomings in addressing and mitigating these unintended health outcomes post-disaster. Data analysis in SPSS used the chi-square test and Student’s t-test.

*Findings:* The study found that the prevalence of waterborne diseases across the CD blocks in Sundarbans was significantly higher in the cyclonic years compared to the non-cyclonic years (t = 6.69), regardless of the seasons. Prevalence of vector-borne diseases was also significantly higher in the cyclonic years compared to the non-cyclonic years (t = 2.55). It was also found that the existing literature lacks detailed accounts of shelter residents’ experiences, illnesses, and pre-existing health issues, particularly addressing the needs of vulnerable populations like women, children, and the elderly.

*Conclusion:* The study highlights gaps in India’s research on evacuee experiences in cyclone shelters, particularly for vulnerable populations like women, children, and the elderly and sick. Future research should focus on primary studies focusing on evacuee experiences, material innovation, and climate-resilient design of cyclone shelters.

## Background

The Bay of Bengal (BOB) region, particularly the Sundarbans, is a hotspot for cyclones and tidal surges, intensified by climate change. Over 140 years (1877–2016), there has been a shift in median cyclone landfall locations throughout time, with high-impact zones detected in northern Odisha and the Sundarbans region of West Bengal [1]. From 1961 to 2020, the region bore the brunt of 132 cyclonic storms, with the Indian Sundarbans notably affected by 13 of them [2]. Between the years 2009 and 2024, the Indian Sundarbans faced about 14 cyclones of varying intensity (Appendix) [3]. Such weather extremes usher in devastation, displacing populations, wrecking infrastructure, and interrupting essential services. Consequently, epidemics of water-related or vector-borne diseases (VBDs) have become rampant, presenting immense health threats. The people of the Sundarbans grapple with environmental dilemmas compounded by poverty, limited water, inadequate sanitation, and prevalent diseases [4].

Cyclones and tidal surges are violent natural events with profound consequences on human health, both directly and indirectly [5]. Injuries, death, wound infections, and asphyxiation are direct ramifications caused by debris, flooding, or collapsed structures. Indirect health impacts involve disease outbreaks stemming from disrupted water and sanitation systems which lead to acute health conditions like waterborne diseases (WBDs) such as typhoid, diarrhea, and acute diarrheal diseases. The disruption of water and sanitation systems can create breeding grounds for disease-carrying vectors like mosquitoes, leading to an increased incidence of VBDs, for example, dengue fever, malaria, and chikungunya [6]. Cyclones and tidal surges may result in crop damage and disruption in food supply chains may lead to food shortages, resulting in malnutrition and its associated long-term health consequences (e.g., stunting and wasting) [7]. The traumatic experiences associated with cyclones and tidal surges, including loss of homes, livelihoods, and loved ones, can lead to mental health issues such as post-traumatic stress disorder (PTSD), anxiety and depression, respiratory conditions, and chronic diseases [7, 8]. Besides, post-disaster recovery is hampered by roadblocks, power cuts, and disruption of healthcare services. Limited access to medications and related stressors can impact the management of chronic diseases such as diabetes, hypertension, and cardiovascular conditions [7].

Cyclone Aila, which hit India, making landfall on May 25, 2009, with sustained wind speeds of 110 km/hour, was essential in highlighting the importance of enhancing disaster preparedness and fostering creative strategies for constructing cyclone shelters to create resilient communities. Cyclone Aila caused the deaths of over 200 individuals in Bangladesh and the eastern Indian state of West Bengal. According to reports, the storm resulted in a minimum of 500,000 individuals being displaced and rendered homeless [9]. Government of West Bengal figures indicate that a total of 6.77 million individuals were impacted, and 137 fatalities occurred in the severely afflicted districts of North and South 24 Parganas in West Bengal [10]. After the occurrence of ‘Aila’ in 2009, there was a noticeable decline in the health of children as a result of insufficient water and sanitation services [11–13]. Post-Aila, a remote region in the Sundarbans called Pakhirala hamlet, was investigated for an outbreak of diarrhea. This investigation revealed a significant adverse impact on health caused by Aila [4, 11]. In addition, the healthcare system in the region saw substantial difficulties with Cyclone Amphan, which struck on May 20, 2020, with wind gusts reaching 165 km/hour. The storm reached a maximum surge of 5 meters [14], causing a flooding incident, while waterborne illnesses spread rapidly as a result of water contamination [15]. West Bengal experienced 86 deaths due to Amphan, causing a minimum of 1.02 trillion Indian rupees in damages, directly affecting 70% of the state’s population. Odisha also saw four fatalities, with 4.4 million individuals affected in varying degrees. The storm destroyed 500 residences and 4,000 livestock, primarily poultry [16]. To mitigate the effects of cyclones on vulnerable communities, India adopted the concept of public cyclone shelters (a concept developed in Bangladesh in the 1960s) to provide secure shelter to vulnerable communities during cyclonic storms and storm surges following a devastating storm in Andhra Pradesh in 1977 [17, 18]. The realization that the structural conditions of shelters deteriorate rapidly under saline environmental conditions led to the concept of multi-purpose cyclone shelters (MPCs) to ensure proper maintenance [19]. Adaptation mechanisms like officially designated cyclone shelters in the Sundarbans to counter cyclones and their effects on community health were only done after the rampage of Aila. As the name suggests, these shelters are meant to be used for multiple purposes, such as schools, public distribution systems, rural animal husbandry units, or veterinary clinics [20]. However, the inadequacy of cyclone shelters in general, including both foundational and recent constructs, is exacerbated by population pressures and scarcity of water, sanitation, and medical provisions [21]. As per the National Cyclone Risk Mitigation Project, the accommodation capacity of MPCSs built with the support of the World Bank ranges from 450 to 1,000 individuals [22].

After the aftermath of Aila in 2009, the higher mortality rate in the impacted areas was attributed to the lack of secure shelter infrastructure in the coastal villages. Hence, the central and state governments made the appropriate decision to construct MPCSs in high-risk areas of three coastal districts in West Bengal. Under the Prime Minister’s National Relief Fund (PMNRF), 50 MPCs were sanctioned in North 24 Parganas (20), South 24 Parganas (15), and Purba Medinipur (15) districts of West Bengal at an approved outlay of Rs.138.65 crores (USD 16.59 million) [23]. The construction work of all 50 cyclone shelters has been completed, and they were handed over to the local authority/State Government of West Bengal in March 2014 [24]. After this (in 2015), 150 MPCSs were mandated to be built in these same three districts under Stage II (NCRMP-II) of the National Cyclone Risk Mitigation Project (NCRMP-II). Additionally, as of June 2022, 146 MPCSs have been built in these districts [25]. A total of 118 MPCSs were built in North 24 Parganas (n = 43) and South 24 Parganas (n = 75) and handed over to the authorities [25]. These MPCSs comply with safety measures such as proper signage, road maintenance, and waste management. They also provide hooks for cattle and boats, comply with water usage and quality standards, maintain cleanliness and sanitation, and provide standard toilets for the disabled population. They also offer electrical safety measures, lightning arresters, emergency evacuation plans, fire extinguishers, and first aid kits [25]. While essential, cyclone shelters alone cannot mitigate disasters wrought by climate change in the Sundarbans. Holistic strategies like upgrading disaster alerts, optimizing shelter placements, and assessing insurance feasibility are vital [26].

Hence, this study aims to gather evidence on the effects of MPCSs on the health outcomes of the population in the Sundarbans. Specifically, we are interested in studying the influence of MPCSs on epidemic-prone diseases that occur after cyclones and tidal surges.

## Aims and Objectives

**Aim:** To investigate the impact of MPCSs on the health outcomes of the Sundarbans’ residents, particularly in the context of epidemic-prone diseases following cyclones and tidal surges.

**Sub-Aims:**

1. To evaluate the direct and indirect health impacts on communities residing in cyclone shelters after cyclonic and tidal surge events in the BOB region.
2. To assess cyclone shelters’ effectiveness and potential shortcomings or gaps in addressing and mitigating these unintended health outcomes post-disaster in the BOB region.

### Study area

The Indian Sundarbans spread across two districts of North and South 24 Parganas, including 19 CD blocks altogether spread across an area of 9630 sq. km [27]. It is located in one of the world’s largest deltas created by the Ganges–Meghna–Brahmaputra rivers draining into the BOB, between 21°40ʹ to 22°40ʹ north latitudes and 88°03ʹ to 89°07ʹ east longitudes [28]. This deltaic ecosystem supports diverse mangrove species, flora and fauna, and human communities.

## Approach

### Database

The disease data at the block level for all 19 CD blocks which house an estimated population of 4.6 million (2022) [29] were obtained from the Health Management Information System (HMIS) portal for the time period 2010–2021 [30]. The data, however, were not available for 2009 or the full year of 2021. The socio-economic and amenities data were obtained from the Census of India, 2011 [31] and cyclonic events data were obtained from the International Best Track Archive for Climate Stewardship (IBTracs) [3]. The list of MPCSs was obtained from the Department of Disaster Management, Government of West Bengal [25]. The map bases for the location map were obtained from a living atlas of Environmental Systems Research Institute, Inc (ESRI), India and the maps were prepared in ArcGIS Pro v.2.8, while SPSS v.27 was used for data analysis.

The HMIS Portal and the Census of India provide secondary data on disease occurrence in the Sundarbans region, allowing for the evaluation of direct and indirect health impacts on communities taking shelter in cyclone shelters. The Census of India provides socio-economic and amenities data, which enables finding associations between disease prevalence and factors like population density, access to safe drinking water, toilet facilities, and covered drainage. The IBTracs provides cyclonic events data, enabling comparison of disease prevalence between cyclonic and non-cyclonic periods. The Department of Disaster Management’s list of MPCSs also aids in assessing disease prevalence and vulnerability.

### Conceptual framework

**Figure 1:**
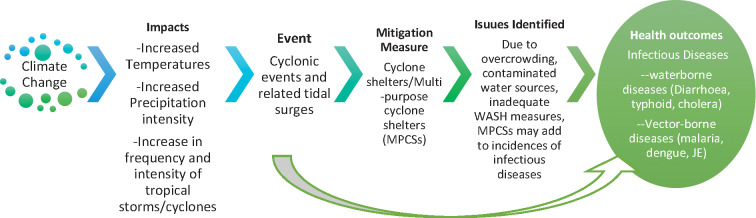
Conceptual framework.

The conceptual framework (Figure 1) represents the link between climate change indicators and increased tropical storms/cyclones, necessitating the construction of cyclone shelters or MPCSs. However, these shelters may face challenges like overcrowding, contaminated water sources, and insufficient water, sanitation and hygiene (WASH) measures, leading to increased occurrences of infectious diseases like WBDs (e.g., diarrhea, typhoid, cholera) and/or VBDs (e.g., malaria, dengue).

For sub-aim 1:

- The disease data were aggregated and categorized into cyclonic and non-cyclonic years to look into the variation of disease occurrence. Also, the blocks were categorized into coastal and inland blocks and into different categories of vulnerability to environmental hazards. This paper used indicators of exposure caused by natural hazards (such as elevation, slope, rainfall deviation, drainage density, proximity to the coastline, cyclone track density, storm surge, and flood inundation risk), indicators of sensitivity (population density, household density, poverty ratio, child dependency ratio, agricultural land, cropping intensity), and adaptive capacity indicators (literacy rate, workforce participation, population in permanent houses, road length, doctors/lakh population, electricity, and banking facilities) [32]. This comprehensive inter-block vulnerability status of the Indian Sundarbans was based on the composite vulnerability index calculated in a study [32], using the average credited rank values of two methods (analytical hierarchy process and principal component analysis). These average scores were then classified into five categories (very high, high, medium, and low), ranging from lowest (<2.06) to highest (>4.56), using the equal interval method [32].
- Given the fact that in India, the months of May-June and October-November are considered to be cyclone season, a year has been divided into three time points for the sake of analysis, i.e., pre-cyclone (January–April), cyclone (May-June and October-November), and post-cyclone (July–September, December). The rationale for including the period from August to December is that there have been cyclone incidences in the BOB in recent years, including October (2022), November (2019), and December (2023). Sundarbans was affected by cyclones from 2013 to 2015 and 2018 to 2021. Hence, these are considered cyclonic years while 2011, 2012, 2016, and 2017 are considered non-cyclonic years when the depressions that formed in the BOBdid not develop into cyclones or our study area was not affected by cyclones. The average prevalence of disease has been calculated and mapped in ArcMap v 10.8.2. Disease prevalence is categorized into five groups (very low, low, moderate, high, and very high) based on the natural breaks criteria given by Jenks [33].
- Chi-square tests and a Student’s t-test were applied to the data to check for associations and differences in disease prevalence across coastal vs. inland blocks, pre-cyclonic vs. post-cyclonic periods, and cyclonic vs. non-cyclonic years.

For sub-aim 2: A literature review was conducted using the following search terms in PubMed, yielding 62 results: ((cyclones [Title/Abstract] OR “cyclone shelters” [Title/Abstract] OR “tidal surges” [Title/Abstract]) AND (infectious disease [Title/Abstract] OR diseases [Title/Abstract] OR disease outbreaks [Title/Abstract])). Similarly, Google Scholar was searched using the following keywords “cyclone shelters” AND India AND “disease outbreaks”, resulting in 122 results. The time limit was placed from 2000 to 2024. Since climate change has been focused on more in recent decades, we chose to restrict our search to this time. The gray literature and newspaper articles were also searched. Reference lists of relevant studies were also searched for any missing papers.

## Findings

### Status of MPCS and prevalence of diseases in the Sundarbans

**Figure 2:**
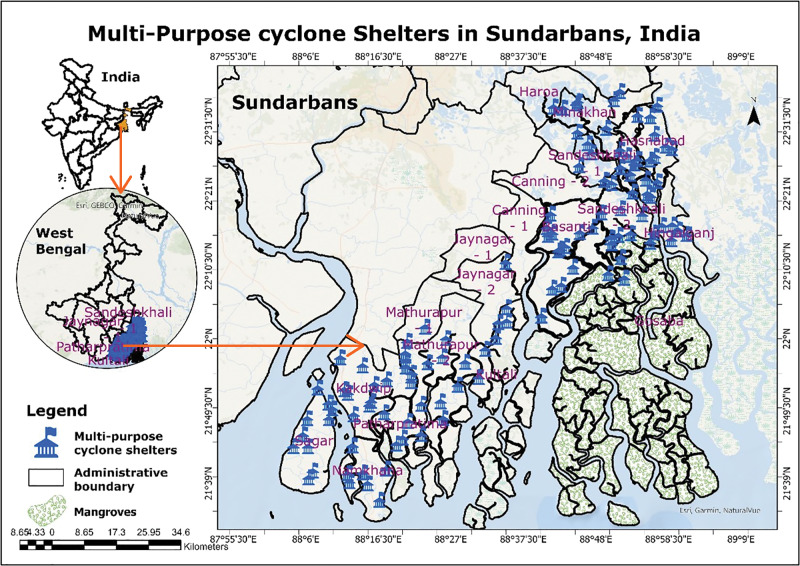
Location of the study area.

There are currently 118 MPCSs in the study area which were handed over to the government in 2023 (Figure 2). The number of cyclone shelters is significantly higher (p < 0.05) in coastal blocks (n = 70) compared to inland blocks (n = 40) (χ2 = 16.13). The number of cyclone shelters is not significantly associated with the population density of the blocks nor with the vulnerability of the blocks.

**Figure 3:**
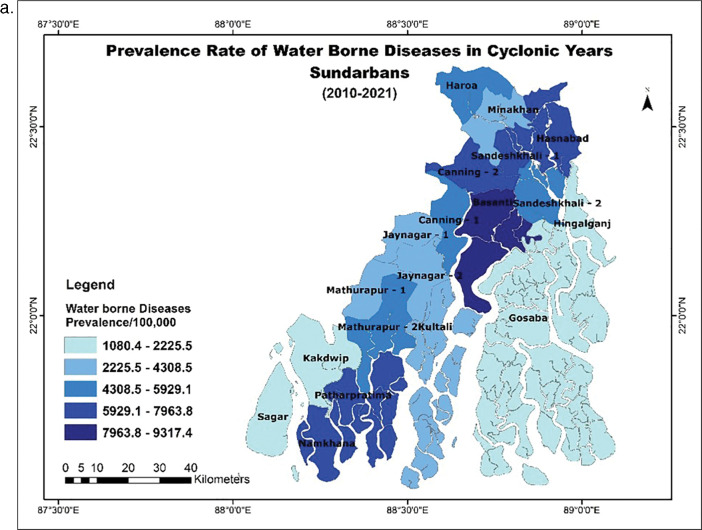

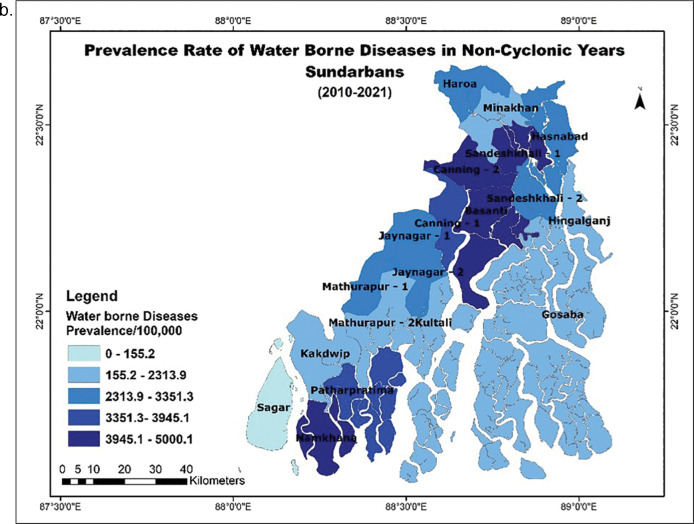
**(a)** Average prevalence rate of waterborne diseases in cyclonic years, Sundarbans (2010–2021) and **(b)** Average prevalence rate of waterborne diseases in non-cyclonic years, Sundarbans (2010–2021).

Our secondary data analysis revealed that the prevalence of WBDs across the blocks in Sundarbans is significantly higher (p < 0.001) in the cyclonic years (Figure 3a) compared to the non-cyclonic years (t = 6.69) (Figure 3b). However, there is no significant difference between the prevalence of disease between the coastal and inland blocks or the vulnerability of the blocks (Appendix I).

**Figure 4:**
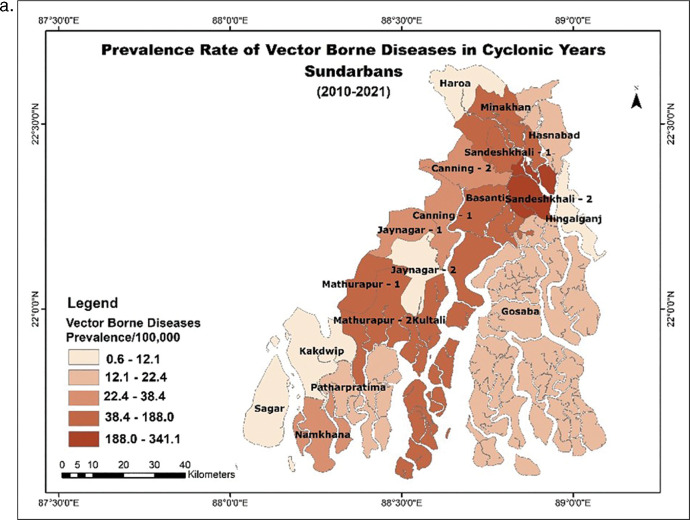

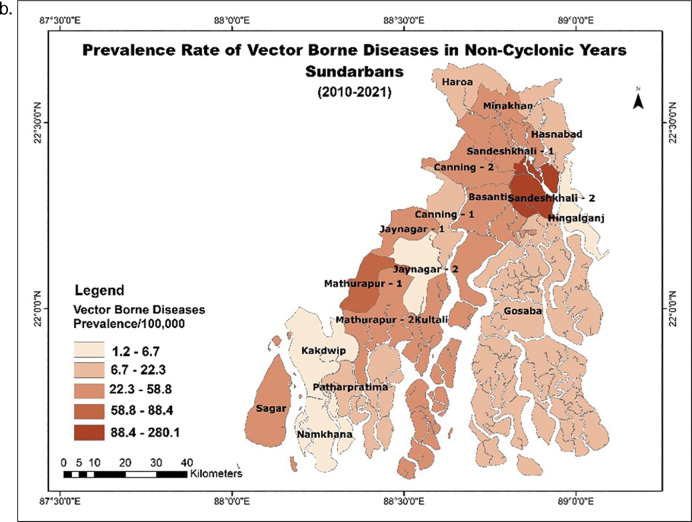
**(a)** Average prevalence rate of vector-borne diseases in cyclonic years, Sundarbans (2010–2021) and **(b)** Average prevalence rate of vector-borne diseases in non-cyclonic years, Sundarbans (2010–2021).

The prevalence of VBDs across the blocks in Sundarbans is also significantly higher (p < 0.01) in the cyclonic years (Figure 4a) compared to the non-cyclonic years (t = 2.55) (Figure 4b). In this case, also, there does not exist any significant difference between the prevalence of disease in the coastal and inland blocks or with the vulnerability of the blocks (Appendix II).

There does not exist any significant difference in the prevalence of these diseases at the three times in a year: pre-cyclone (January-April), cyclone (May-June and October-November), and post-cyclone (July–September, December). Further, at these three points, no significant difference exists between the average prevalence of diseases across the coastal and inland blocks in the cyclonic years.

**Figure 5:**
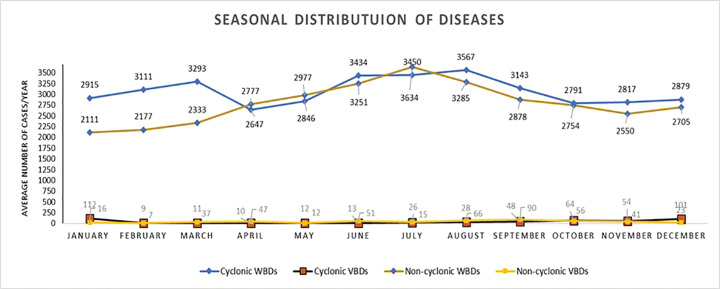
Seasonal distribution of diseases (2009–2021).

Also, as seen from Figure 5, the cases of WBDs show an increase in the month of June (perhaps a couple of weeks after a cyclonic event) for cyclonic years, while a higher peak is noticed in July for non-cyclonic years. However, a clear pattern of VBD distribution could not be established.

The prevalence of a disease is not significantly associated with the percentage of households having access to safe drinking water, toilet facilities, and covered drainage facilities. The coastal blocks (Namkhana, Basanti, Patharpratima, Gosaba, Kakdwip, Kultali, and Sagar) have a significantly lower (p < 0.01) percentage of households having access to safe drinking water (F = 9.44) and covered drainage facilities (F = 8.79) compared to inland blocks (Sandeskhali I, Canning II, Canning I, Haroa, Hasnabad, Sandeskhali II, Mathurapur I, Jayanagar II, Jayanagar I, Mathurapur II, Minakhan, and Hingalganj).

### Insights from literature

The literature obtained was broadly divided into two themes: (i) studies assessing the health impact of cyclones on human population in the region and (ii) studies assessing the design, role, and management techniques of MPCSs. Additionally, the evacuation behaviors of the evacuees and their life experiences, especially of women, were also reported. Findings from these two themes are presented as and , respectively, and summarized here.

The impact of cyclones on the health of the inhabitants of Sundarbans seems to be majorly focused on the literature after the severe cyclonic storm Aila in 2009. Certainly, this cyclone paved the path for a focused study on climate change-induced environmental hazards in the Sundarbans region and the discussion on relief measures, coping mechanisms, loss suffered, and the impact of such hazards on the ecosystem as well as human lives [34, 35]. In recent times, from 2010 to 2024, more literature has been published on the topic of studying the districts of the east coast of India [6].

Diarrhea and cholera (which causes severe diarrhea) were the most commonly reported diseases post-cyclones; other infectious diseases like vector-borne diseases, viral fevers, enteric fevers, and food poisoning were also listed [6], along with skin diseases and typhoid [36]. One study conducted in the post-Phailin period in Odisha reported 9 (n = 50) cases of snake bites in children (1 month–14 years). Out of these, seven studies were conducted in India and one from Bangladesh (Appendix III).

In terms of literature on cyclone shelters and their impact on the health of the people, there was almost no literature available for India. Some studies in India in recent decades have looked into the suitability of cyclone shelters during the cyclone period. A study in Bangladesh was conducted on the lived experience of cyclone shelter seekers, especially looking at it from the lens of gender [37]. Out of the 11 studies identified from the 2009 to 2024 time period, five were conducted in Bangladesh, and five were in India, out of which three of them covered areas of the BOB coast (one from West Bengal, three from Odisha, one from Tamil Nadu and one focusing on Andhra Pradesh, Odisha, and Gujarat) (Appendix IV). The newspaper articles focused on the path, weather conditions, impact, and rescue details of the cyclones. However, descriptions of the effectiveness or shortcomings of the cyclone shelters were missing.

## Discussion

Cyclone shelters have been identified as one of the most common measures to mitigate cyclone-associated risk in many parts of South Asia. Their efficacy is shown to be better in comparison to early warning systems, since the effectiveness of early warning systems can be compromised if individuals cannot find safe shelter or evacuate in time, particularly in vulnerable areas [38]. These play a crucial role in safeguarding people’s lives, particularly in coastal areas that are highly vulnerable to cyclonic storms, especially severe storms like Amphan, Yaas, and Remal. The MPCSs are believed to reduce morbidity and mortality. However, a study in 2022 found these shelters not safe enough for people, damaged structures pointing to the lack of proper maintenance, and inadequate capacity in terms of the population of the areas they are built in, like in the case of Odisha [19]. The increase in infectious diseases after a cyclone may be due to contamination of water and food sources due to cyclones and related tidal surges leading to flooding. Overcrowding, limited hygiene practices, and inadequate ventilation due to higher population density can also be factors causing infectious disease transmission after cyclones [19, 39]. Another study in Bangladesh focused on the experience of women in shelters and found that women felt unsafe in these shelters. These women were found to suffer from physiological injuries, psychological stress, genito-urinary infections, and post-natal health issues. Consequently, they developed post-traumatic stress disorders, panic attacks, and anxiety problems causing a decline in their overall well-being and impacting their future lives.

These issues may not be the intended consequences of MPCSs but are something to be clearly thought about. Selected studies in India reported concerns about overcrowding, waterlogging, sanitation, and lack of hygiene in the cyclone shelters, which are similar to the findings from studies conducted in Bangladesh [40, 41].

The current case study has identified the following gaps from the literature:

- No study in India focuses on the experiences of the evacuees in the MPCSs of the Sundarbans. Recent studies conducted in Odisha have discussed the suitability of cyclone shelters as safe spaces during cyclones.
- The needs of especially vulnerable populations like women, children, and the elderly and sick are not comprehensively addressed in the existing literature. Understanding the experiences of this vulnerable population in cyclone shelters is crucial for addressing their unique challenges and ensuring their safety. This knowledge can guide shelter design, service provision, training, and community engagement, fostering inclusivity and support.
- Existing literature fails to shed light on the impact of overcrowding in cyclone shelters on public health in general, and for women and children in particular. Overcrowding can lead to several public health issues, such as the spread of infectious diseases, limited access to basic hygiene facilities, and mental health-related stressors. Addressing this critical gap is essential for understanding the public health implications of shelter management.
- Existing literature lacks detailed accounts of the experience of people who stayed in the shelters, their illnesses or other pre-existing health issues. Understanding these personal experiences will certainly help to improve future preparedness strategies.
- There is a major gap in studies on the prevailing cyclone shelter management system and its effectiveness. Research on shelter management, including coordination, stakeholder involvement, resource allocation, and service availability, is crucial for developing a robust, effective, and efficient management system for cyclone shelters.

Future areas of research should evaluate the physical condition, maintenance practices, and adherence to building codes and standards of existing MPCSs. Experiences of the evacuees should be explored to find out workable solutions to the problems of overcrowding, hygiene, and the safety of women, children, the elderly and the sick, and a comprehensive structural analysis of shelters should be conducted using advanced engineering techniques, simulation, and modeling studies to assess shelters’ responses to cyclonic events. Additionally, innovative and locally sourced materials in building these shelters may be explored for making sustainable and climate-resilient MPCSs.

## Conclusions

The study found that while literature focuses on cyclones’ health impacts, it often neglects cyclone shelters’ impact on evacuees’ health. Climate change events and adaptation measures, like cyclone shelters, affect people’s well-being. Proper maintenance and hygienic conditions of these shelters are crucial for the health of those seeking refuge.

Cyclones and tidal surges affect various geographical areas within the BOB region differently. Health concerns can be influenced by factors such as the distance from the coast, altitude, and the level of infrastructure development. Examining the geographical disparities in health outcomes can assist in identifying regions with higher risks and informing specific actions, such as the strategic positioning and structure of cyclone shelters.

A key challenge in this case study was the lack of climate change and health-related data, especially disease-related data at the block level. Data for 2009 and earlier, as well as updated data for 2021–2023, were unavailable, leading to overlooked insights. Additionally, there is no secondary data on the incidence of infectious diseases among MPCS evacuees; this information can only be obtained from field research.

Effective adaptation research in the country requires two vital things. The first is to make health data accessible and share it in a timely and fully transparent manner so that the research community can use evidence-based interventions and policies for the promotion of health. Second, collaboration between researchers, government entities, and communities should be enhanced for the betterment of citizens’ health. Hence, it can be concluded that only providing shelter, toilets, and first aid is insufficient. Regular maintenance and hygiene during the evacuees’ stay, unbiased distribution of essential services like food and medicines, and segregated safe spaces for women and children are also necessary. Additionally, facilitating research in these areas requires collaboration, building the technical capacity of researchers, and ensuring the accessibility of health data.

Hence, to enhance cyclone shelter management, the following recommendations are suggested:

- Proper maintenance: Develop and adhere to precise and thorough maintenance schedules for cyclone shelters, involving the community in the process.
- Training and capacity building: Provide comprehensive training on maintenance techniques and sanitation protocols to the local authorities and community volunteers.
- Provision of sufficient resources and supplies: Ensure MPCSs have the necessary resources and supplies to meet their maintenance and hygiene needs.
- Routine inspections: Conduct routine inspections to detect and address any maintenance or hygiene concerns.
- Monitoring and feedback mechanisms: Establish a system to monitor and track maintenance and hygiene practices and incorporate feedback procedures.

## Data Availability

The study is based on secondary data obtained from publicly available sources. Specific details about the data sources, links and licensing information have been provided in the methods section and listed in the references as well.

## Role of Authors

Sajda Khatoon undertook conceptualization along with Martin W Bloem and Pranay Lal. Sajda Khatoon and Paramita Bhattacharya undertook secondary data curation. Data visualization, writing original draft was undertaken by Sajda Khatoon. Paramita Bhattacharya worked on the methodology, analyzed the data. Nirmalya Mukherjee provided the working resources and software. Pranay Lal and Martin W Bloem also validated the findings. Pranay Lal, Martin W Bloem, and Nirmalya Mukherjee supervised, reviewed, and provided constructive feedback for the improvement of the manuscript. All authors reviewed and agreed to the final manuscript.

All authors had access to the data and a role in writing the manuscript.

## Appendices

**Appendix I:** Cyclone affecting the Indian Sundarbans (2009–2024).

S. NO.	YEAR	CYCLONES AFFECTING THE INDIAN SUNDARBANS	CATEGORY (IMD) [42]	WIND SPEED IN KM/H	SAFFIR–SIMPSON HURRICANE SCALE [43]
1	2009	Bijli	Cyclonic Storm	75	1
2	May 25,09	Aila	Severe Cyclonic Storm	110	2
3	2013	Viyaru	Cyclonic Storm	85	1
4	Oct12, 13	Phailin	Extremely Severe Cyclonic Storm	215	5
5	Oct12, 14	Hudhud	Extremely Severe Cyclonic Storm	185	4
6	July 30, 15	Komen	Cyclonic Storm	75	1
7	2018	Titli	Very Severe Cyclonic Storm	150	4
8	May 03,19	Fani	Extremely Severe Cyclonic Storm	215	5
9	Nov 01, 19	Bulbul	Very Severe Cyclonic Storm	140	4
10	May 20, 20	Amphan	Super Cyclonic Storm	150–240	5
11	May 01, 21	Yaas	Very Severe Cyclonic Storm	130–140	4
12	Oct 5, 22	Sitrang	Cyclonic Storm	85	1
13	May 12, 23	Mocha [44]	Extremely Severe Cyclonic Storm	210–220 km/h gusting to 240	5
14	May 26, 24	Remal [44]	Severe Cyclonic Storm	110–120 km/h gusting to 135 km/h	3

*Sources:*

Indian Meteorological Department (IMD). Cyclones. NDMA, GOI. 2024. https://ndma.gov.in/Natural-Hazards/Cyclone National Hurricane Center, Central Pacific Hurricane Center, NOAA. Saffir-Simpson Hurricane Wind Scale. 2024. https://www.nhc.noaa.gov/aboutsshws.php?os=app&ref=app India Meteorological Department. Preliminary Report Cyclones. 2024. https://rsmcnewdelhi.imd.gov.in/report.php?internal_menu=MjY=

**Appendix II:** Table showing block wise vulnerability, geographic location, diseases’ prevalence, and other variables of association.

AVERAGE YEARLY DISEASE PREVALENCE RATE PER 100,000													
S. NO.	DISTRICTS	BLOCKS	VULNERABILITY	GEOGRAPHIC LOCATION	WBDS (CYCLONIC YEARS)	WBDS (NON-CYCLONIC YEARS)	VBDS (CYCLONIC YEARS)	VBDS (NON-CYCLONIC YEARS)	HHS HAVING TOTAL SAFE DRINKING WATER (%)	HHS HAVING TOILET FACILITIES (%)	HHS HAVING CLOSED DRAINAGE SYSTEM (%)	NO. OF MPCSS	POPULATION DENSITY (POPULATION PER SQ. KM)
1	**South 24 Parganas**	Sagar	Very High	Coastal	1080	155	12	44	90.3	86.9	0.3	10	541
2		Namkhana	Very High	Coastal	7964	4874	27	2	99.3	72.8	0.2	10	560
3		Kakdwip	Very High	Coastal	1717	1623	11	1	97.6	59.5	1.3	9	863
4		Patharpratima	High	Coastal	6614	3945	22	15	99.3	59.4	0.1	12	474
5		Kultali	Very High	Coastal	2880	1558	136	39	100	32.3	0.2	7	263
6		Mathurapur I	Medium	Inland	4309	2952	95	88	99.7	40.8	0.9	0	1503
7		Mathurapur II	Very High	Inland	5363	2314	188	35	99.4	54.9	0.9	5	771
8		Jayanagar I	Medium	Inland	4029	2860	28	36	98.9	49.9	2.1	0	1287
9		Jayanagar II	Very High	Inland	3669	2877	9	3	97.4	32.4	1.1	0	2279
10		Canning I	High	Inland	5929	3745	32	14	99.5	59.1	3.2	0	1427
11		Canning II	High	Inland	7244	4975	38	52	98.7	57.4	0.6	0	719
12		Basanti	Very High	Coastal	9317	4712	138	42	98.2	40	0.5	12	504
13		Gosaba	High	Coastal	2118	1984	19	22	96.4	75.3	0.3	10	93
14	**North 24 Parganas**	Hingalganj	Medium	Inland	2225	1494	1	7	86.2	74.3	0.6	11	615
15		Hasnabad	Low	Inland	7406	3289	17	16	91.4	63.1	1	5	1318
16		Haroa	Low	Inland	5258	3351	2	20	97.2	80.7	1.7	0	1310
17		Sandeskhali I	Medium	Inland	6670	5000	132	59	98.2	64.1	1.6	11	870
18		Sandeskhali II	High	Inland	4727	3030	341	280	98.9	55.9	0.7	12	812
19		Minakhan	Medium	Inland	3478	1570	114	42	97.2	80	1.9	4	1263

HHs = Households; WBDs = Waterborne diseases; VBDs = Vector-borne diseases; MPCSs = Multi-purpose cyclone shelters

**Appendix III:** Literature on the impact of cyclones on human health in the BOB region.

NO	AUTHOR	CYCLONE STUDIED	STUDY AREA	POPULATION	OBJECTIVES	METHOD	DISEASES	FINDINGS
1	Giribabu, D., Muvva, V. R., Joshi, N. K., & Rao, S. S. 2021	Multiple	Eastern coast districts, India	none	To evaluate the impact of WASH interventions, the prevalence of disease epidemics during cyclones in India from 2010 to 2018, and the correlation between cyclones and disease outbreaks	Used meteorological parameters, disease data from the Integrated Disease Surveillance Program, etc. from 2010 to 2018 to compile an inventory of disease outbreaks during cyclones	**Infectious disorders such as acute diarrheal diseases, malaria, viral fevers, enteric fever, and food poisoning** have repeatedly been reported during cyclonic occurrences and lasted for up to two weeks after the cyclone	The effectiveness of Clean India Mission was evident in recent storms like Ockhi, Titli, and Gaja, with a notable decrease in disease outbreaks
2	Kabir. R, Khan. H, Ball. E & Caldwell. K., 2016	Sidr & Aila	Amtali Upazila of Barguna District, cyclone Aila affected Koyra Upazila, Khulna District, **Bangladesh**	2 FGDs with max 10 members each	To assess the impact of cyclones Sidr and Aila on the inhabitants of coastal Bangladesh	A qualitative study using primary data collection: Focus Group Interview was followed by a thematic analysis	It found an increase in **waterborne illnesses like diarrhea, typhoid, and skin diseases** due to contaminated water sources	
3	Mishra. S., Ram Kumar. T., & Biswas.AK., 2016	Phailin (Odisha)	Berhampur, Odisha, India	children aged 1–14 months divided in three groups: G1 study group (n = 50); g2 control groups (n = 25); G3 control group (n = 29)	To analyze the incidence of poisoning cases in children before and after Phailin, in order to assess the impact on the pediatric population	1. This retrospective study used hospital data from Maharaja Krishna Chandra Gajapati Medical College and Hospital, Berhampur, Odisha. 2. Analyzed three groups: post-Phailin Study group, pre-Phailin Control group B, and post-Phailin Control group C in the following year. 3. Data Analysis chi-square tests or t-tests	**Snake bites** in children 1 month to 14 years	18% (N = 9/50)
4	Mazumdar S, Mazumdar PG, Kanjilal B, & Singh PK., 2014	Aila	Hingalganj, Gosaba and Patharpratima, Sunadarbans, India	809 individuals from 179 households	To evaluate the effects of Cyclone Aila on households and the subsequent strategies employed to deal with the situation in three severely impacted sub-districts, namely Hingalganj, Gosaba, and Patharpratima	Cross-sectional household survey	Not investigated occurrence of specific health impacts	Aila has caused significant damage to 54% of households’ assets, leading to a lack of financial support and access to government relief, institutional credit, and mortgage or distress pawning. Typical strategies include borrowing from informal lenders, family and friends, and relying on household income
5	Bhunia R, & Ghosh S., 2011	Aila	Sundarbans, India	57 cases and 171 controls	The study aimed to ascertain the causative agent and origin of the disease outbreak, and to suggest strategies for its containment	Matched case control study: data on reported diarrhea cases was collected from January 2007 to May 2009, stool specimens for probable cases were tested, interviews with cases conducted and water tested for contamination	**Cholera**	1,076 cases resulting in 14 deaths. Attack rate:44/10,000
6	Panda, S., Pati, K. K., Bhattacharya, M. K., Koley, H., Pahari, S., & Nair, G. B., 2011	Aila	East-Medinipur in West Bengal, India	39 samples	The study investigates the increase in diarrhea cases following the AILA storm in East Medinipur	Primary data were collected through field visits and stakeholder conversations, while secondary data were analyzed over three years. Laboratory examinations involved rectal swabs and chi-square tests to evaluate temporal patterns and disparities in antibiotic usage	**Cholera** (severe form of diarrhea)	The bacterium Vibrio cholerae was detected in 54% (n = 21/39) of the collected samples, providing evidence of a widespread occurrence of cholera within the community. Incidence of diarrhea increased following Cyclone Aila in June 2009, particularly in the Haldia and Egra subdivisions. The Vibrio cholerae isolates were found to be antibiotic resistant but were sensitive to norfloxacin and azithromycin. Haldia had the highest prevalence incidence of diarrhea, with an attack rate of 9 per 1000
7	Bhattacharjee S, Bhattacharjee S, Bal B, Pal R, Niyogi SK, Sarkar K., 2010	Aila	Pakhirala village of the Sundarbans, a coastal region of South 24 Parganas, India	37 stool samples were tested in the lab	The study investigated a watery diarrhea outbreak in Pakhirala village, Sundarbans region, analyzing morbidity, causative agents, and clinical results, comparing cases in Pakhirala and other villages	Stool samples were collected from cases in Pakhirala village	**Diarrhoea**	June 5–July 20, 2009, 91% (n = 3592) compared to 70% (n = 28,550) in other villages
8	Chhotray G. P et al., 2002	Super cyclone	Odisha, India	107 rectal swabs collected from hospitalized diarrhea patients	To analyze causative agents of cholera outbreak in cyclone-hit areas in Orissa	Molecular analysis, including PCR assays and ribotyping, was conducted on V. cholerae strains. Antimicrobial susceptibility testing and genetic characterization	**Diarrhoea** agent: *Vibrio cholera*	77.57% (n = 83/107) diarrhea cases

**Appendix IV:** Literature related to cyclone shelters in the BOB region.

NO	AUTHOR	CYCLONE STUDIED	STUDY AREA	POPULATION	OBJECTIVES	METHOD	FINDINGS
1	Anburaja Durai et al, 2023		Tamil Nadu, India		Study focuses on the structural design of a circular cyclone shelter for high-intensity cyclones	Analyzed existing shelters using STAAD-Pro software	**Circular cyclone shelters are more effective** and suitable for environmental conditions compared to existing rectangular shelters Circular shelters are **found to withstand extreme wind loads and provide better protection during cyclones**, making them a recommended choice for establishment along the east coast of Tamil Nadu
2	Jaiswal. A, et al., 2022		India (AP, Odish, Gujrat) & Bangladesh	NA	To consolidate diverse management techniques implemented in the South-Asian region, specifically in Bangladesh and India, for MPCSs	Thematic or content analysis to report patterns in shelter management It includes a semi-systematic review of existing literature to map theoretical themes and identify gaps	The shelter **should be effectively managed and equipped for use during disaster** All the **equipment provided in MPCSs must be in working condition** The **shelter should be well maintained**, to be usable when required the most
3	Chowdhury et al, 2022		Bangladesh		Examines the first-hand experiences of females residing in cyclone shelters in Bangladesh and analyzes their physical and mental well-being as individuals seeking refuge in these shelters	Max van Manen’s methodological approach to hermeneutic phenomenology was adopted	In order to be prepared for potential cyclones, multiple cyclone shelters have been built. However, a significant number of the coastal population, particularly women, are reluctant to utilize these shelters Women found themselves in a disadvantaged position in the shelter, which was similar to the experiences of women across the globe
4	Kanjilal & Bhandari, 2022		Digha, West Bengal		1. to understand the operation and upkeep of the current MPCS (Multi-Purpose Community Spaces) 2. To evaluate the level of connectivity between each existing MPCS and the adjacent villages in Ramnagar-I and II. 3. To determine the amount of space provided per individual in each MPCS, in comparison to the government’s stipulations	Conducting a household survey The local government departments have furnished data regarding the various structural characteristics of the cyclone shelters The SRTM DEM dataset from 2014 was utilized to obtain the land’s elevation. The road networks are obtained from the ISGP website	Storm shelters in local areas are not distributed fairly, with essential facilities like kitchens and toilets often non-functional due to insufficient maintenance The lack of understanding among the population and transportation issues makes people reluctant to relocate. Official rules suggest each MPCS can only accommodate 50–60% of the local population, but these shelters can only accommodate 300 people, which is insufficient to meet the needs of the large number of vulnerable people seeking refuge
5	Hadi et al, 2021	Amphan, Aila, Sidr, Gorky	Bangladesh	210 participants from seven coastal districts; Males-135, Females-75	Analyze factors that influence the decision to evacuate to cyclone shelters in Bangladesh over the past 30 years, focusing on Cyclone Amphan (2020) and historical cyclones Gorky (1991), Sidr (2007), and Aila (2009)	The study used a mixed-method approach to analyze data from literature reviews, household surveys, and phone interviews	Studies on the decision to evacuate in Bangladesh’s coastal regions following Cyclone Gorky reveal that fear of property loss leads to partial evacuations, environmental cues analysis before evacuation, and finding sanctuary in neighboring houses Cyclone shelters are poorly distributed and lack amenities. Despite advancements in disaster response infrastructure, evacuation rates have not increased Among the recommendations are gender-responsive projects to provide safe surroundings for people seeking asylum and risk-based planning for shelters
6	Mohanty. S., et al., 2021	Amphan	Odisha, India	2 KIIs	To outline the challenges encountered in managing shelters during cyclones amidst the COVID-19 pandemic in Odisha	Literature, reports and direct interviews of field professionals and practitioners	The study reveals that storm shelters are typically converted into schools and managed by local communities or elected entities However, new concerns include urging vulnerable groups to evacuate, converting shelters into COVID-19 facilities, addressing cleanliness and safety requirements, and accommodating high-risk populations
7	Dash & Walia, 2020	Phailin’ (2013) Hudhud (2014) Titli (2018)	India	5–30 FGD members across four FGD groups including people from various occupations	To examine the precise role of MPCSs that they aim to perform after four cyclones (Phailin, Hudhud, Titli & Fani) during 2013–2019. It assesses the decision-making criteria involved in choosing an MPCS and gains experiential knowledge of evacuees taking shelter in the MPCSs	Qualitative and follows multiple case study method Primary data collected using Group Discussion and Interviews	Feeling unsafe inside the shelter, especially at peak cyclone intensity. Poorly maintained shelter buildings leading to rainwater leaking inside the rooms and Damage to shelter buildings to varying extents: cracks on building walls, removal of fittings and iron doors, etc. Moreover, the total number of individuals housed in every MPCSs represents only a meager fraction of the inhabitants in the districts. Shortfall in CS capacity: Available shelter capacities are only 7.3% of the population in vulnerable regions
8	Das.S., 2018	Phailin	Odisha, India	320 households from coastal shelter villages in Odisha, India.	The study aimed to investigate the evacuation behavior of individuals who are susceptible to cyclones and are prone to evacuating	A two-stage random sampling technique to choose 320 households from shelter villages located along the shore at a distance of 1–2 km from the coastline was selected A household survey was conducted and interviews conducted in the native language Data analysis: descriptive statistics and logistic regression	“Evacuees from all districts encountered difficulties during their stay in shelters. The issues reported include limited space (33), insufficient food supply (16), inadequate water access (13), absence of basic bathroom facilities (09), absence of prepared meals (5), electrical problems (3), and rainwater seepage”
9	Seo. N, 2017	All cyclones in NIO from 1990 to 2015	Bangladesh	All cyclones in NIO from 1990 to 2015	The study aims to assess the effectiveness of the Cyclone Shelter Program (CSP) in reducing fatalities caused by cyclones, specifically focusing on its impact on high storm surges and intense winds	Negative binomial regression model to quantify the impact of the CSP on reducing deaths caused by cyclones	Given the same level of storm surge, this program is estimated to reduce fatalities by 75%
10	Haider. Z, & Ahmed. F.,2014		Morelgonj and Sarankhola upazila under Bagerhat district of Bangladesh	144 respondents, 12 each from each of the 12 shelter catchment areas	Identify income-generating activities for cyclone shelters in Bangladesh	Focus Group Discussion (FGD), survey, and stakeholder consultation	Each shelter has a capacity ranging from a minimum of 500 −2500 persons The entire number is insufficient to provide meaningful support for the vulnerable people affected by the disaster The current cyclone shelters in the coastal region lack adequate provisions for lighting, water, sanitary facilities, and separate rooms for women
11	Mallick. B, 2014	Aila	Southwest coastal area of Bangladesh	308 respondents from selected villages	To assess the necessity of constructing additional Community Shelters (CSs) in the future or explore other approaches for community-based cyclone disaster management	Household survey and focus group discussions A stratified random sampling procedure to select respondents from villages affected by Cyclone Aila Focus group discussions were conducted with survivors to analyze mobility patterns and social mapping of institutional supports	The study found that only 15.5% of early warning participants brought their family members to a community shelter, while 25.3% sought refuge in a nearby residence During the storm, two-thirds of respondents were at home or in a nearby residence, with a significant proportion at educational facilities, religious institutions, or boats
